# Students’ perceptions of their STEM learning environment

**DOI:** 10.1007/s10984-023-09463-z

**Published:** 2023-04-12

**Authors:** Nicole Fairhurst, Rekha Koul, Rachel Sheffield

**Affiliations:** School of Education, Curtin University, GPO Box U1987, Perth, WA 6845 Australia

**Keywords:** Classroom emotional climate, Integrated STEM learning, STEM education, STEM learning environments, Student perceptions, Teacher–student interactions

## Abstract

Australia’s economic need for innovation has led to Science, Technology, Engineering and Mathematics (STEM) education becoming an essential investment for the future. This study utilised a mixed-methods approach involving a pre-validated quantitative questionnaire together with qualitative semi-structured focus groups with students across four Year 5 classrooms. Students provided their perceptions of their STEM learning environment and their interactions with their teacher to identify factors influencing their engagement for pursuing these disciplines. The questionnaire comprised of scales from three different instruments: Classroom Emotional Climate, Test of Science Related Attitudes, and Questionnaire on Teacher Interaction. Several key factors were identified through student responses, including Student Freedom, Peer Collaboration, Problem Solving, Communication, Time, and Preferred Environments. 33 out of possible 40 correlations between scales were statistically significant, but eta^2^ values were considered low (0.12–0.37). Overall, the students expressed positive perceptions about their STEM learning environment, with Student Freedom, Peer Collaboration, Problem Solving, Communication and Time appearing to impact their perceptions of STEM education. Three focus groups with a total of 12 students identified suggestions for improving STEM learning environments. Implications from this research include the importance of considering student perceptions when measuring the quality of STEM learning environments, as well as how facets of these environments can impact student attitudes towards STEM.

## Introduction

Education frequently refers to the STEM acronym as the partial or full integration of the separate disciplines of Science, Technology, Engineering and Mathematics, including a focus on twenty-first Century competencies (Koul et al., 2018; Timms et al., 2018). Research evidence suggests there is a need to advance STEM education across Australia in order to ensure international economic competitiveness (Education Services Australia, 2018; Hudson et al., 2015; Office of the Chief Scientist, 2013). One of the key reasons for this drive is the decline in enrolments and performance within STEM education (Education Council, 2015; Education Services Australia, 2018). Caplan et al. (2016) explain that, whereas studies have been conducted within high schools, research indicates the need to engage students prior to the ages of 11–14 years to ensure longterm interest in pursuing these disciplines.

STEM education and the development of critical STEM skills are essential for Australia’s future of economic success, particularly when faced with unknown working conditions due to innovation and technology (Caplan et al., 2016; Honey et al., 2014; Marginson et al., 2013; Timms et al., 2018). The Foundation for Young Australians (2017) identified that occupations requiring these skills have risen by 70% and involve higher pay than those which don’t. Additionally, the World Economic Forum (2017) highlighted that automation and COVID-19 will have impacts on working conditions and that around 85 million jobs could be displaced by these changes; however, 97 million new roles will replace these jobs, requiring additional sets of skills. The World Economic Forum's (2017) top 15 skills for 2025 are outlined in Table 1 and refer to the skills that will be essential for the future workforce. Therefore, the development of STEM competencies from an early age is crucial to building a workforce with the capacity to undertake these new roles. These skills are referred to later within the Results and Discussion sections to highlight links between STEM education and the needs of the industry.

**Table 1:** World Economic Forum (2017) top 15 skills for 2025

Rank	Skill
1	Analytical thinking and innovation
2	Active learning and learning strategies
3	Complex problem-solving
4	Critical thinking and analysis
5	Creativity, originality and initiative
6	Leadership and social influence
7	Technology use, monitoring and control
8	Technology design and programming
9	Resilience, stress tolerance and flexibility
10	Reasoning, problem-solving and ideation
11	Emotional intelligence
12	Troubleshooting and user experience
13	Service orientation
14	Systems analysis and evaluation
15	Persuasion and negotiation

Because of the need to inspire the young generation’s enthusiasm for STEM education, it is important to determine effective strategies or circumstances that target engagement across the integrated disciplines. Learning environment research is an extensively researched field that has been built upon for decades. The learning environment can be described as the psychosocial and emotional dimensions of a classroom that are identified from the perspective of a student and/or educator, including relationships, perceptions and attitudes (Fraser, 2012). The use of extensively validated questionnaires to measure perceptions within these environments is an established practice (Koul et al., 2018). Teachers utilise their learning environments to convey their expectations, directly impacting student perceptions of learning areas (Watt, 2016). Therefore, it is critical for researchers to determine which characteristics of STEM learning environments have positive or negative impacts on these perceptions in promoting engagement and aspiration.

## Background

### STEM education

The exact definition of STEM education is widely debated; however, its meaning is important for educators to be able to implement it successfully (Blackley & Howell, 2015; Rosicka, 2016; Timms et al., 2018). While the drive to teach STEM through an integrated approach is more prevalent (Thomas & Watters, 2015) in Western education, there are still arguments that it should be delivered through its individual content areas. Rosicka (2016) believes that content isolation does not position students to understand how concepts relate to the world outside the classroom, therefore making integration more relevant. This approach supports students to fail authentically, reflect on their attempts, and develop positive mindsets for solving problems (Rosicka, 2016). Nadelson and Seifert (2017) describe integrated STEM education as the delivery of contextual problems through which students experience a combination of the concepts and content, positioning them to practise skills and knowledge that are naturally required. This process is more reflective of real-world industry experiences. For this paper, STEM education refers to the full or partial integration of Science, Technology, Engineering and Mathematics, with a focus on twenty-first century competencies (Koul et al., 2018; Timms et al., 2018). This is reflective of the authentic approaches discussed in the literature which position students to engage with real-world integrative experiences to develop transversal skills.

Murphy et al. (2019) refer to student attitude towards STEM education and aspiration as ‘STEM dispositions’, and state that positive self-perceptions across these disciplines are essential for sustained engagement. They also claim that developing these positive perceptions early is integral to ensuring interest (Murphy et al., 2019). Through a survey of 15 000 public school students, Wiebe et al. (2018) found that children as young as elementary age have already begun to form attitudes and associations between their life, academic experiences, and potential career pathways. Marginson et al. (2013) explain that some students have negative perceptions of STEM education because they believe that this type of study is something that students with ‘talent’ undertake, rather than being accessible to everyone through hard work. As positive perceptions of STEM are integral to student engagement, it is essential to determine the factors associated with STEM education which influence student perceptions.

### Learning environments

This study builds on the learning environment research conventions in which student perceptions are key psychosocial factors influencing student learning (Koul & Fisher, 2005). Walberg (1976) advocates the use of students’ perceptions to assess learning environments because students are quite capable of perceiving and weighing up stimuli and rendering predictively valid judgements of the social environments of their classes. Students’ perceptions of their classroom learning environment have been shown in a multitude of studies over the past decades to reliably predict affective and cognitive outcomes (Fraser, 2012, 2014; Fraser et al., 2020). Learning environment research identifies the psychosocial and emotional dimensions of a classroom from the perspective of educators and/or students, including relationships, attitudes, perceptions, and the nature of the environment (Fraser, 2012). Fraser (2012) determined that students, who spend significant time within classrooms, have perceptions which are essential to consider. A number of studies have found correlations between student attitudinal outcomes, cognitive outcomes and learning environments (Fraser, 1986, 2012), indicating that student perceptions are critical to their engagement with education. Further, other elements of learning environments, such as Classroom Emotional Climate, Teacher–Student Interactions and Attitudes, relate strongly to a child’s academic achievement and social-emotional growth (Rucinski et al., 2018), and therefore are discussed in following sections in relation to this project.

### Classroom emotional climate

The Classroom Emotional Climate (CEC) refers to the extent that positive emotions and student comfort are promoted within a classroom (Brackett et al., 2011). Positive CEC includes showing care and concern; listening to and acting upon student perspectives; fostering peer cooperation; and showing an awareness of children’s academic and emotional needs (Hamre & Pianta, 2007). Contrasting negative environments include teachers who can threaten, disrespect or humiliate, or have poor emotional connections with students (Reyes et al., 2012). Neutral CEC also exists, with students likely to be unsure about how to approach their teacher because of inconsistencies in their behaviour (Reyes et al., 2012). Reyes et al. (2012) explain that classrooms which are reflective of positive CEC are more likely to have students who are engaged, enthusiastic and academically successful.

CEC was an important construct to this research project because it is a predictor of academic success and engagement (Reyes et al., 2012; Rucinski et al., 2018). Within Australia, the need to improve student academic success and engagement is significant for being able to meet the predicted workforce demands of industry (Barkatsas et al., 2018). Therefore, measuring CEC within STEM learning environments can provide aspects of this construct that positively impact a student’s aspiration to be successful and pursue STEM education (Fraser et al., 2020). Additionally, seeking elements of this construct which have negative implications could also assist with supporting students to engage positively.

### Teacher–student interactions

Teacher–Student relationships are an important construct within learning environments because an educator’s motivational style has the potential to directly influence the engagement of a student (De Loof et al., 2021). They can be defined as the type and strength of the personal relationship between the teacher and student (Fraser & Walberg, 2005), and are determined by associated interactions. Research has revealed an empirical link between student achievement, student attitude, and their relationship with their teacher (Fraser & Walberg, 2005). Further, teachers play a crucial role in influencing career and study decisions, including their capacity to nurture a love of STEM education (Office of the Chief Scientist, 2013). For these reasons, teacher–student relationships were measured alongside CEC in this study to further understanding of how these constructs impact student perceptions of STEM education.

A team of researchers (Brekelmans et al., 1990; Wubbels et al., 1985, 1991; Wubbels & Levy, 1993) extrapolated the seminal interpersonal behaviour research of Leary (1957) to develop the Questionnaire on Teacher Interaction (QTI) for gathering students’ perceptions of their teachers’ behaviours. This development led to a strong focus in classroom learning environment research on focusing teacher–student interactions (Fraser & Walberg, 2005). Brekelmans et al. (1990) investigated teacher behaviour in classrooms from a systems perspective, adapting a theory on communication processes (Watzlawick et al., 1967) that assumes that the behaviour of the teacher is influenced by the behaviour of the students and this, in turn, influences student behaviour. Circular communication processes develop which not only influence behaviour, but determine it as well.

**Fig. 1:**
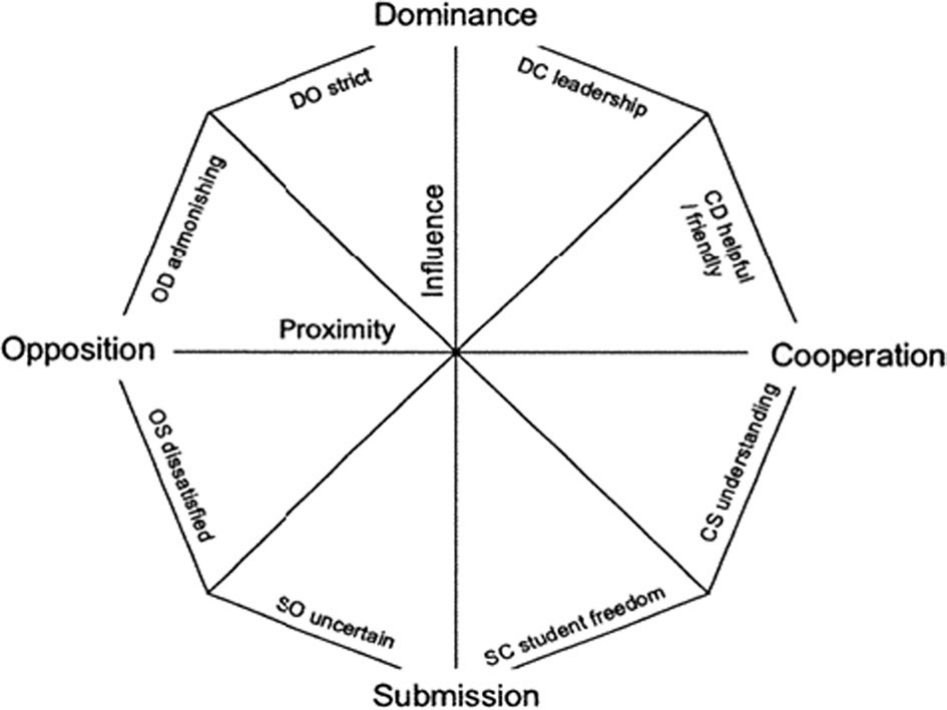
Model for interpersonal teacher behaviour (den Brok et al., 2005)

The QTI measures interpersonal behaviour (Fig. 1) through Influence (Dominance–Submission) and Proximity (Opposition–Cooperation) (den Brok et al., 2005). This was expanded into an eight-sector model (Fig. 1) and the QTI was developed to assess student perceptions of these eight aspects of behaviour. This instrument measures the positive behaviours of Leadership, Helping/Friendly, Understanding, Student Responsibility/Freedom and the negative behaviours of Uncertain, Dissatisfied, Admonishing and Strict. The initial instrument was developed for use in secondary schools and later Goh and Fraser (1997) developed and validated a version for primary schools. In line with the precedents set by Fraser et al. (2020), this study only used positive set of scales of the QTI instrument, which directly contribute to the teacher–student interactions dimension of this research project.

### Attitude to STEM

**Fig. 2:**
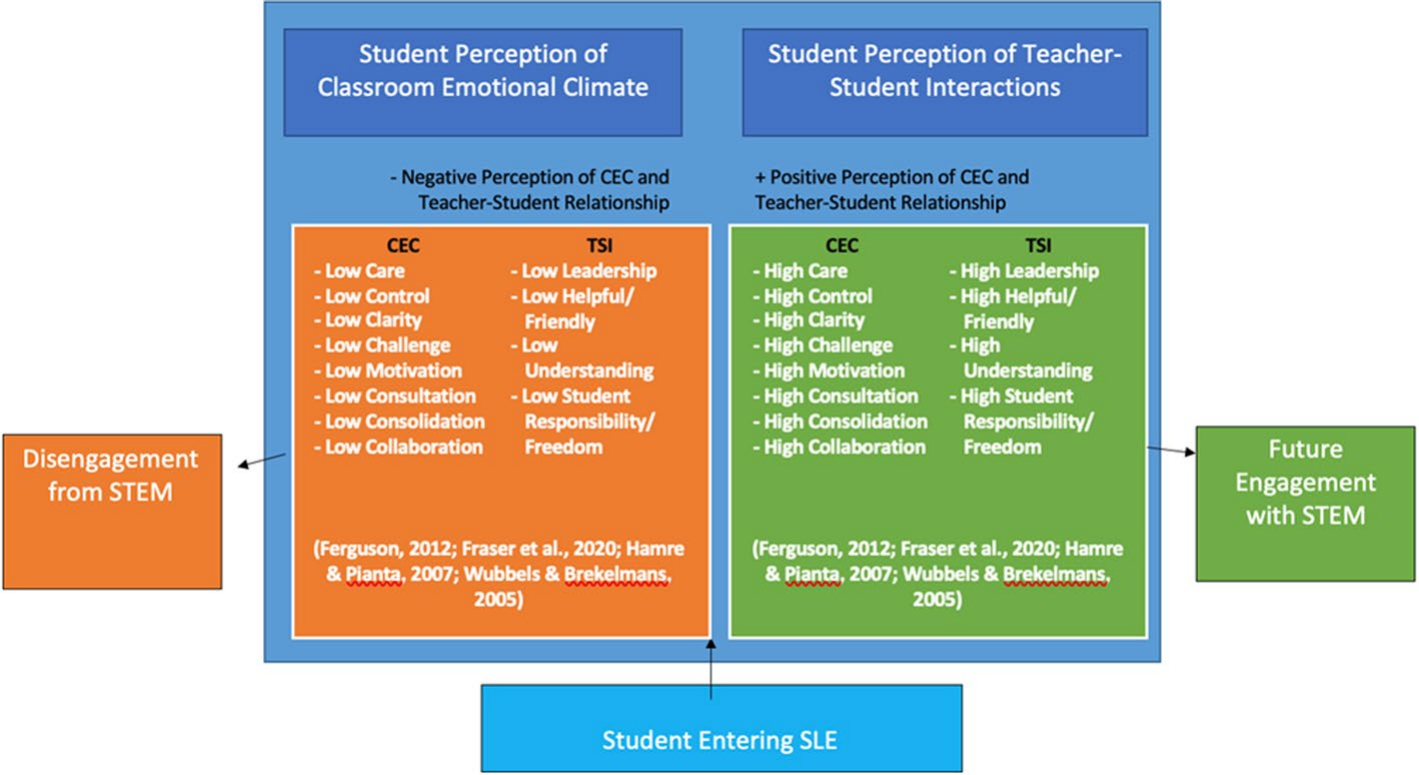
Model of hypothesised relationships between students’ perceptions of teacher–student interactions, classroom emotional climate and attitude towards STEM

The attitudes to STEM education dimension for this study included modified scale of Attitude of Scientific Inquiry from the widely-used Test of Science Related Attitudes (TOSRA) (Fraser, 1981). This 70-item seven-scale instrument targets middle- and high-school students and specifically measures their attitude towards science education (Fraser, 1981). In this study, we used a scale specifically focussed on student perceptions of their current attitude towards STEM education as used by Fraser et al. (2020), in order to investigate possible characteristics of the learning environment that influence student attitudes. A model of the hypothesised relationships is provided in Fig. 2.

### Defining STEM learning environments

Currently, STEM learning environments have not been adequately researched (Fraser et al., 2020) and, therefore, little is known about them. It was important in this study that the definition of STEM learning environment was a combination of the integrated STEM education approach. For this reason, it was defined as an integrated learning context which utilised at least two of the STEM disciplines, while students practised their 21st Century competencies to solve problems (Yang & Baldwin, 2020), and perceptions of psychosocial and emotional dimensions were measured from the perspective of an educator and/or student.

## Objectives

The overarching aim of the study was to determine student perceptions of the quality of their STEM learning environment and their interactions with their teacher, as well as how this impacted their attitude towards STEM education. An additional aim was to investigate student preferred perceptions of their environment in an attempt to determine characteristics which may improve engagement. The specific objectives of the research reported in the paper were to:

- Further validate a combination of scales from three pre-validated questionnaires for assessing upper-primary student perceptions of their STEM learning environment and their interactions with their teacher;
- Investigate associations between the three constructs of (1) the Classroom Emotional Climate, (2) student Attitudes to STEM and (3) the quality of teacher–student interactions;
- Investigate gender differences in perceptions;
- Further investigate students’ preferred perceptions of their STEM learning environment, as well as potential characteristics which are conducive to improved engagement;
- Use thematic analysis of focus groups to identify themes to facilitate understanding of student perceptions of STEM learning-environments.

## Methods

### Design

This research utilised a mixed-methods case study approach to determine the perceptions of upper-primary students on their STEM learning environments and their interactions with their teacher. Upper-primary education was a focus because it is at this early age when children begin to form their career aspirations (Wiebe et al., 2018). This project firstly utilised the pervasive approach within learning environment research involving using a quantitative questionnaire to measure student perceptions of their classroom emotional climate, their interactions with the STEM teacher, and attitudes to STEM. Fraser et al. (2020) suggests that combining quantitative and qualitative methods when implementing questionnaires assists with explaining findings and provides greater detail. To elicit further understanding from students, semi-structured focus groups were conducted with a smaller number of students to collect further details about these perceptions, as well as their preferred teacher behaviours. Ethics approval for this study was granted by the Human Research Ethics Committee of our University.

### Sample

This project was conducted at a co-educational independent school in Perth, Western Australia within Year 5 classrooms. The context was chosen because of its reputation for successfully implementing STEM education, therefore making it suitable for measuring the a quality of the STEM learning environment and investigating the impacts on student perceptions. Additionally, teachers of the four classes collaborated on their projects, so the students shared similar experiences. While the teachers did not always refer to the acronym ‘STEM’, they utilised the integrated approach as defined for this style of education.

### Quantitative methods

The quantitative information was collected through the questionnaire which was comprised of scales from three tools and used in a previous study (Fraser et al., 2020). The first instrument measuring Classroom Emotional Climate (CEC) was based on the Classroom Assessment Scoring System (CLASS) and the Tripod 7 C’s student perception survey (Ferguson, 2012; Fraser et al., 2020; Hamre & Pianta, 2007). Its eight scales of Care, Control, Clarity, Challenge, Motivation, Consultation, Consolidation, and Collaboration were adopted from Fraser et al. (2020). The second section, which measured Attitude to STEM, was adapted from the Attitude to Science scale from the Test of Science Related Attitudes (TOSRA) (Fraser, 1981). The final section measured teacher–student interactions and was based on the positive scales of the Questionnaire on Teacher Interaction (QTI) (Fraser et al., 2020). The four scales are Leadership, Helpful/Friendly, Understanding, and Student Responsibility/Freedom (Wubbels & Brekelmans, 2005). While originally designed for high school classrooms, both the TOSRA and QTI have been validated within primary school classrooms, making them appropriate for our study (Goh & Fraser, 1997; Koul et al., 2018). The instrument was firstly piloted through six focus groups, and then validated through the use of Exploratory Factor Analysis (EFA) and Confirmatory Factor Analysis (CFA) in a junior high school setting, with a reasonable sample size of 658 participants in a separate study (Fraser et al., 2020). Functional validation of this instrument within upper-primary students was then completed through this project.

Prior to students completing the questionnaire, the researchers discussed STEM experiences with the participants, including what STEM is and projects or experiences completed at school. During implementation, to ensure understanding and to allow opportunities for questions, the researcher read each item to the students as they responded using a 5-point Likert scale. Additionally, the reliability estimates suggest that students had the capacity to respond to the items as intended.

The questionnaire was administered to 100 students (46 male; 54 female) across four Year 5 (10–11 years old) classrooms with(student ratio 23:24:26:27). All students who had provided consent, and were present on the day, participated in the study. The sample size was impacted by COVID-19, because some students had not returned to school after it had been shut down during the pervious term.

Quantitative analysis was conducted through a range of strategies. Data from the questionnaire was inputted into an Excel spreadsheet, and then processed using IBM SPSS version 27 Statistics Software. Firstly, descriptive statistics were generated for means and standard deviations for the data set. *T*-testing was used to compare genders and Analysis of Variance (ANOVA) compared means between classrooms. Correlations and muiltiple regressions were used to determine significant relationships between variables. Additionally, alpha reliability, eta^2^ and Pearson’s correlation were reported.

### Qualitative methods

A sample of 12 students was purposively selected to participate in semi-structured focus groups; an outline of participant selection is shown within Table 2. Five male and seven female students were purposively selected,based on their gender and their responses to the Attitudes to STEM items to ensure representation, to determine any differences of opinion between male and female students. We selected 3 out of 5 male students who indicated positive perceptions of STEM education and that they were motivated to pursue further study in STEM. One male indicated negative perceptions and no motivation to pursue further STEM education. The final male student was undecided about STEM education, and indicated no motivation to pursue it further. Three female students were included who indicated positive perceptions of STEM education, and motivation for further STEM learning. Two indicated negative perceptions and no motivation to the field. The final two female students indicated that they were undecided and had no motivation to pursue STEM further. Guiding questions were utilised to help to prompt the students to discuss their thoughts about their STEM learning environment.

**Table 2:** Purposive sampling for qualitative focus groups based on quantitative results

Gender	*N*	Attitude towards STEM		Motivated to Pursue STEM			
Male	5	Positive (≥ 4)	3	Indicated	3	Not Indicated	0
		Negative (2 ≤	1	Indicated	0	Not Indicated	1
		Undecided (2 > < 4)	1	Indicated	0	Not Indicated	1
Female	7	Positive (≥ 4)	3	Indicated	3	Not Indicated	0
		Negative (2 ≤	2	Indicated	0	Not Indicated	2
		Undecided (2 > < 4)	2	Indicated	0	Not Indicated	2

Thematic analysis was applied to the qualitative data. Braun and Clarke (2006) state that this approach, when applied rigorously, can reveal insightful and valid information, and is particularly useful for highlighting similarities and differences of perspectives between participants. Because this project had a focus on differences between male and female perspectives, this approach was particularly beneficial.

A deductive thematic analysis was used for this project because the researcher brought preconceptions and ideas to the coding which developed through the guiding questions. This also allowed connections to be made naturally between the questionnaire and the responses from the semi-structured focus groups. However, any codes or themes that were not linked to the preconceived concepts were used to ensure that the students had voice and that data collection was flexible to these ideas.

During preliminary data examination, transcripts of the focus groups were created by an online professional company for accuracy. The coding process was comprised of a systematic approach to the transcripts, with data points being highlighted and given their initial first codes. These data were then categorised based on collective meaning. The coded data were rearranged into more-refined themes, with some of the data points that reflected more than one category being placed within their ‘best fit’ categories. This process, and the resulting themes, were then reflected upon and reviewed by two colleagues to ensure accuracy and relevance, as suggested by (Braun & Clarke, 2006). From these final themes, data points were examined alongside the quantitative data to further improve reliability.

## Quantitative results

### Descriptive information

Tables 3 and 4 provide descriptive information, namely, mean and standard deviation, for each questionnaire scale. Overall, students indicated positive perceptions of their classroom emotional climate (Table 3). The four classrooms overall indicated an average mean score of 4.07 out of a possible 5 for Classroom Emotional Climate. Control had the highest mean of 4.30 whereas Consultation had the lowest mean of 3.85.

**Table 3:** Scale mean, standard deviation and internal consistency for the scales of STEM CEC

Scale	No of items	Mean	SD	Alpha reliability	Mean correlation	Eta^2^
Care	10	4.15	0.63	0.87	0.65***	0.25
Control	8	4.30	0.55	0.77	0.56***	0.12
Clarity	9	4.02	0.70	0.87	0.67***	0.20
Challenge	9	4.10	0.64	0.83	0.66***	0.23
Motivation	9	4.15	0.71	0.88	0.61***	0.25
Consultation	8	3.85	0.74	0.82	0.62***	0.24
Consolidation	12	3.90	0.71	0.88	0.67***	0.37
Collaboration	9	4.11	0.61	0.84	0.44***	0.22
Average		4.07	0.66	0.85	0.61	0.24

^*^p < 0.05, **p < 0.01, ***p < 0.001 *N* = Students = 100, Classes = 4

**Table 4:** Scale mean, standard deviation, and internal consistency for Attitude to STEM and scales of teacher–student interactions

Scale	No of items	Mean	SD	Alpha reliability	Mean correlation	Eta^2^
Attitude to STEM	10	4.08	0.96	0.97	0.44***	0.31
Leadership	5	4.46	0.59	0.78	0.68	0.16
Helpful/Friendly	5	4.28	0.73	0.80	0.72**	0.22
Understanding	4	4.28	0.87	0.88	0.70**	0.27*
Student Responsibility /Freedom	4	2.47	0.97	0.74	0.46	0.34**
Average		3.91	0.82	0.83	0.57	0.23

^*^p < 0.05, **p < 0.01, ***p < 0.001 *N* = Students = 100, Classes = 4

Teacher–student interactions and attitude to STEM scales also had a reasonably high mean (Table 4). Overall, the four classes indicated a mean of 3.91 out of a possible 5 for their relationship with their teacher. It is particularly interesting to note the low scores which are affecting the mean average for Teacher-Student Interactions from the Student Responsibility/Freedom scale. The students reacted reasonably negatively towards this scale, and further insight was given during the semi-structured focus groups. This scale particularly focussed on the teacher’s tolerance for student behaviour within the classroom, with students indicating that they did not like large amounts of freedom within this area. They indicated that their teachers didn’t allow misbehaviour within their classroom, and they were able to expand on this reasoning further within the focus groups.

### Research objective 1: Questionnaire validation

Validation of the 8 CEC scales (Table 3) and 5 Attitude to STEM and Teacher–Student Interaction scales (Table 4) involved three indices. First, each scale’s internal consistency reliability was ascertained using Cronbach’s alpha coefficient. Second, each scale’s independence (or discriminant validity) was checked using the mean correlation of a scale with the other scales as a convenient index. Third, the ability of each scale to differentiate between different classrooms was tested via ANOVA, with the eta^2^ values reported in Table 3 and 4 indicating the proportion of variance in a scale’s scores attributable to class membership.

Regarding scale independence, the mean correlation of a scale with the other scales ranged from 0.49 to 0.67 for the 8 CEC scales (Table 3) and from 0.44 to 0.72 for the 5 attitude and interaction scales (Table 4). This suggests that questionnaire scales overlap somewhat.

The ANOVA results in Tables 3 suggest that most of the 13 questionnaire scales were able to differentiate between the perceptions of students in different classrooms, with eta^2^ values ranging from 0.12 to 0.37 for CEC and from 0.16 to 0.34 for attitude and interaction scales.

### Research objective 2: associations between classroom emotional climate, teacher interactions and attitude to STEM

Table 5 shows the analysis of associations between the scales of CEC, QTI and Attitude to STEM based on simple and multiple correlation analyses. The simple correlation analysis revealed that all eight scales of CEC were significantly correlated with Attitude to STEM. These correlations were positive and ranged from 0.32 to 0.51. The multiple correlation (*R*) was 0.61 and statistically significant (*p* < 0.001). This strongly supports the conclusion that the nature of the classroom emotional climate strongly influences students’ attitudes towards STEM. To further interpret this relationship, the standardised regression coefficients (β) were also examined. Out of eight scales, regression weights for three scales retained their significance (*p* < 0.01). This means that the scales of Care, Control and Collaboration are independent predictors of individual students’ Attitude towards STEM class. The *R*^2^ value, which indicates the proportion of variance in Attitude towards STEM class that can be attributed to students’ perception of classroom environment, was 38%.

**Table 5:** Associations between STEM CEC, Attitude scales and teacher–student interaction scales in terms of simple correlations (r), multiple correlations (R), and standardised regression coefficient

Scale	Attitude–Environment Associations									
	Attitude towards STEM		Leadership		Helpful-friendly		Understanding		Student responsibility/freedom	
	*r*	d	*r*	β	*r*	β	*r*	β	*r*	β
Care	0.32**	− 0.35*	0.74***	0.42***	0.67***	0.32**	0.71***	0.43***	0.05	0.06
Control	0.48***	0.25*	0.66***	0.12	0.49***	− 0.14	0.44***	− 0.11	− 0.04	− 0.27*
Clarity	0.44***	0.07	0.65***	− 0.11	0.65***	− 0.05	0.65***	− 0.09	0.07	0.01
Challenge	0.43***	0.01	0.67***	0.02	0.68***	0.19	0.63***	0.02	0.01	− 0.34
Motivation	0.48***	0.16	0.80***	0.54***	0.71***	0.42***	0.59***	0.24*	0.02*	0.63***
Consultation	0.42***	0.05	0.51***	− 0.09	0.52***	− 0.15	0.62***	0.01	− 0.001	− 0.09
Consolidation	0.51***	0.30	0.62***	0.15	0.65***	0.30*	0.72***	0.49***	0.09	0.26
Collaboration	0.46***	0.24*	0.32**	− 0.17*	0.36***	− 0.05	0.25*	− 0.24**	− 0.12	− 0.28*
R	0.62***		0.62***		0.59***		0.57***		0.07	
R^**2**^	0.38***		0.38***		0.35***		0.33***		0.01	

^*^p < 0.05,**p < 0.01,***p < 0.001 *N* = 100

Out of the four positive scales of the QTI, the three scales of Leadership, Helping-Friendly, Understanding demonstrated statistically significant correlations with the all the eight scales of the CEC. Student Responsibility/Freedom was significantly correlated only with one scale (Motivation) of the CEC. As mentioned previously, student emotions were not strongly associated with Student Responsibility, and scale of Control had a negative correlation. For the possible 32 associations, only 13 associations were significant (including three negative associations) (see Table 5). The multiple correlations also reflect a similar result. The first four scales have reasonably high regression coefficients; while the Student Responsibility/Freedom has a nonsignificant coefficient. The coefficient of determination (*R*^2^) are reasonable figures for a human behaviour study for the first four scales, which typically fall under 50% because of the unpredictability of people (Frost, 2019).

### Research objective 3: gender differences

Table 6 presents the gender differences in STEM CEC scales. Only the scales of Control and Motivation scales of CEC indicated significant differences (*p* < 0.01) for gender. Females perceived higher levels of Control with their STEM learning environment, and also that they felt more motivated. Interestingly, there were no significant gender differences for Attitude to STEM, even though males did score slightly higher than females (4.14 males to 4.03 females). Additionally, the standard deviation for Attitude to STEM also is reasonably high, showing a greater range in responses than the scales of the other dimensions. Low effect sizes (Gignac & Szodorai, 2016) between 0.01 and 0.26 further confirmed that both genders perceived STEM learning environments similarly.

**Table 6:** Item mean, item standard deviation and gender differences for of STEM CEC and Attitude to STEM scales

Scale	Mean		S D		Difference	
	Male	Female	Male	Female	*t*	Effect size *r*
Care	4.06	4.22	0.66	0.60	0.44	0.13
Control	4.14	4.43	0.63	0.43	5.94**	0.26
Clarity	4.01	4.03	0.72	0.70	0.10	0.01
Challenge	4.02	4.16	0.75	0.53	2.51	0.11
Motivation	4.04	4.24	0.87	0.54	7.20**	0.14
Consultation	3.86	3.85	0.69	0.78	0.37	0.01
Consolidation	3.96	3.85	0.66	0.75	0.07	0.08
Collaboration	4.03	4.18	0.63	0.59	1.19	0.12
Attitude to STEM	4.14	4.03	1.01	0.92	0.33	0.06
Average	4.02	4.11	0.74	0.65	2.02	0.10

^**^p < 0.01 *N* = Total 100 Students, Male = 46, Female = 54

Table 7 reports gender differences in scales of QTI. The scales of Leadership and Student Responsibility/Freedom both indicated statistically significant gender differences in students’ perceptions (*p* < 0.05). Overall, there were minimal gender differences in student perceptions in terms of effect sizes of between 0.03 and 0.14, which can be considered small (Gignac & Szodorai, 2016).

**Table 7:** Item mean, item standard deviation and gender differences for teacher–student interaction scales

Scale	Mean		S D		Difference	
	Male	Female	Male	Female	*t*	Effect size *r*
Leadership	4.38	4.53	0.68	0.49	4.61*	0.13
Helpful/Friendly	4.16	4.37	0.82	0.64	3.78	0.14
Understanding	4.30	4.25	0.74	0.98	2.55	0.03
Student Responsibility/Freedom	2.51	2.43	1.12	0.84	4.09*	0.04
Average	3.84	3.90	0.84	0.74	3.76	0.09

^*^p < 0.05 *N* = Total 100 Students, Male = 46, Female = 54

Overall, students indicated positive perceptions of their Classroom Emotional Climate, Attitude to STEM and their relationships with their teachers. Because of the quality of the STEM learning environment selected for this study, this is a constructive result. In particular, the highly rated Attitude to STEM scale was a good indication of students’ positive perceptions. The STEM CEC questionnaire was appropriate for measuring these dimensions and yielded good reliability scores. While there were minimal differences between males and females, there were some statistically significant results that provided some insight into gender perspectives within this context.

## Qualitative results

### Research objective 4

The semi-structured focus groups gave greater insight into the perceptions of the students collected through the questionnaire. Overall, students outlined many positive characteristics of their STEM learning environment and how it impacted on their attitude towards STEM education. They were also able to provide their preferred perceptions about what could improve their STEM learning environment.

Thematic analysis is a qualitative research method that can be widely used across a range of epistemologies and research questions. Braun and Clarke (2006) observed that this method is useful for identifying, analysing, organising, describing, and reporting themes found within a data set. All data generated were transcribed and read iteratively to locate concepts being represented by the data. All three researchers in the team independently analysed the data and only themes that were identified in all researchers’ analyses or were accepted for inclusion.

Open coding procedures as delineated by Corbin and Stauss (2008) were utilised and involved continually asking questions such as “Which category does this incident/word/phrase allude to?” and “What are the similarities or differences between the two emerging concepts?” Words/themes/and other data pieces alluding to a particular theme were colour coded. Processes of bundling, grouping similar units, and deletion of synonymous units were utilised to arrive at final categories as delineated in the research findings.

All three authors independently conducted the thematic analysis to arrive at the data themes. The independently generated themes by the authors revealed a high degree of agreement. The data pieces were revisited collaboratively to discuss the disagreements and to develop a consensus on the themes. Focus group discussion transcripts responses were shared with the school principal, who identified themes emerging from the data that could be matched with themes derived by the research team.

Thematic analysis identified seven themes which assist in the understanding of student perceptions of their STEM Learning Environments, as well as perceived preferred environments (see Table 8). Each theme was then further broken into sub-categories that represented different aspects of the theme. Themes 1–6 all included comments, attitudes and feelings about what students were currently experiencing within their STEM learning environments. The final theme, Preferred Environments, included any data points about possible improvements for STEM learning environments. The following sub-sections report results of the thematic analysis.

**Table 8:** Sub-categories of themes derived from student focus group interviews

Theme	Sub-category
1. Student Freedom	1. Boundaries
	2. Choice
2. Peer Collaboration	1. Grouping
3. Problem Solving	1. Teacher Support
	2. Peer Support
	3. Trial and Error
4. Communication	1. Noise
	2. Teacher Control
5. STEM Learning	1. Emotions
	2. Understanding/Misconceptions
6. Time	1. Limitations
7. Preferred Environments	1. Hands On
	2. Environment
	3. Choice
	4. Technology
	5. Peer Collaboration

### Student freedom

The first theme that emerged from the semi-structured focus groups was Student Freedom, which is a particularly interesting theme and insight from the students. It was broken into the sub-categories of Boundaries (items related to teacher setting boundaries) and Choice (items relating to student choice, such as choosing how to solve a problem). Students did not respond well when their classroom was free from boundaries and structures related to behaviour and control; however, students discussed positive freedoms when given options about their learning. Students were comfortable when the teacher gave them boundaries but sought choices that sat within these boundaries. For example, if students were given an integrated STEM project to complete, they might have a choice about optional topics within the scope of their learning or about how to present the final product of their learning.

### Peer collaboration

The second theme to develop was Peer Collaboration, which closely relates to a range of STEM skills, and was only given the one sub-category of Grouping. Students described how they were given ample opportunities to work in teams and the ways in which they formed their learning groups. They explained that their teacher would sometimes choose their groups, allow them to choose groups, or occasionally group them at random. Interestingly, students were able to explain why they thought that their teachers did this, because they knew they were more productive when grouped by an adult. Additionally, they also noted that they performed better when in mixed-gender groups, even though they said that they wouldn’t choose these groups if given the option:

*2.1.2.8: Sometimes our teacher picks our groups. If you do not like that. But sometimes she just lets us cause um.*

*Interviewer: So, do you think that is most of the time? She lets you choose?*

*2.1.2.10: Yeah.*

*2.1.2.11: Um, yeah, she – [teacher] gives us relative, like, um. In our groups, we can really choose who we want. But yeah, she normally says there has to be like, a split gender. ‘Cause otherwise, you just have whole groups of all girls and whole groups of all boys.*

*Interviewer: And what would you prefer?*

*2.1.2.13: Um, I like the split. It gives different perspectives normally.*

*Interviewer: Perspectives coming from the?*

*2.1.2.15: I would probably prefer to have an all-girl group but I think you do work better when you have like, different genders.*

### Problem solving

Problem Solving was the third theme, with the sub-categories of Teacher Support, Peer Support and Trial and Error. Again, this theme has many connections to a range of essential STEM competencies required for a successful future workforce. Teacher Support was discussed within all three of the focus groups, with students being able to outline how they felt very supported during the problem-solving process. They were able to describe how their teachers created a balance between support and challenge through prompting (as opposed to giving answers) and how difficult the learning was. Peer Support was also discussed and highlighted the cooperative nature of the learning environment where students were able to seek answers from each other prior to approaching a teacher for support. While this was the case, they mentioned that they knew that, if they couldn’t find an answer from a peer, their teacher would be happy to guide them. Regarding Trial and Error, the final sub-category for the Problem Solving theme, students discussed being given multiple opportunities to solve problems. Students also indicated that they felt happy that they were able to trial a range of methods, including their own ideas, to try and solve problems. They noted that it allowed them to experience success and improve:

*3.1.1.5: You can get other friends down or the teacher might just let it try and, try and let it [work] out itself, and if you still cannot get it, she will come down and help.*

*3.1.1.6: They [the teacher] give us a sudden urge to like try to find another idea and go around the problem and find a new solution.*

*Interviewer: Great. And how does that make you guys feel?*

*3.1.1.10: Um, better because we know we have something to work with.*

*3.3.1.1: Like, I cannot give up so I can revise how it could be better and what can you – you can improve.*

*Interviewer: How does it make you feel when you do that?*

*3.3.1.3: Uh, happy I guess.*

*Interviewer: Makes you happy?*

*3.3.1.5: Makes us more inspired, so it can like, make more, uh, ideas and better ideas.*

*3.3.1.6: Ah yeah. Just giving advice.*

*3.3.1.7: Just like you are [inaudible], some actual help.*

*3.3.1.8: Yeah, that you help you and use the result to generate ideas.*

#### Communication

The fourth theme was Communication, which was broken into the sub-categories of Noise and Teacher Control. Within this research project, communication was seen as an essential element of skills within STEM learning environments, because of the importance of having the capacity to spread and share new knowledge competently to others. The focus within this theme was the opportunities students were given to communicate and present their knowledge. The Noise sub-category related to the control that the teacher had over the learning environment. While students frequently noted how they had opportunities to work cooperatively or collaboratively to problem solve, they also noted that their teachers could ask them to reduce their noise, even when it was relevant to their projects. It is interesting to note the student perspective on this situation. It is likely teachers ensure that noise stays at a productive level so that there are still supportive boundaries in place for the students to prevent a disruptive environment. The Teacher Control sub-category related to teacher decisions about how students would communicate their knowledge. Students generally reported that they were given guidelines, and this usually meant that all groups were presenting in the same way, but the presentation styles changed for different projects. Students were able to list posters, iMovie, performances, websites, typed reports and speeches as different ways in which their teachers asked them to communicate their knowledge to others.

### STEM learning

The fifth theme was STEM Learning, which was divided into sub-categories of Emotions and Understanding/Misconceptions. This first sub-category centred around emotional experiences within SLEs, whereas the second focussed on student understanding of STEM education. The data indicated, overall, that students were experiencing positive and engaging learning within their SLEs and that their perceptions of STEM were positive. Interestingly, students were not always able to define exactly what STEM education is, but they were able to name learning projects that they had completed and explain how these were STEM learning tasks.

### Time

The sixth theme was Time, with the sub-category of Limitations. This related primarily to the balance between giving the students enough opportunities to problem solve and complete tasks and ensuring that the mandatory curriculum requirements were being met. This is frequently a dilemma for teachers and can be a difficult balance. One of the key points made by students was that they identified that sometimes projects were spread out over weeks or a term, and other times they could be spread out over a single day. They remarked that they preferred projects that went over a day rather than spread between other curriculum subjects, which is interesting but not always possible. Additionally, while they explained that they were given ample opportunities for problem solving, they still felt they didn’t always have enough time to complete their work. As time is a complex factor within classrooms, it can be difficult for both students and educators to navigate this delicate balance.

### Perceived preferred learning environments

Perceived Preferred Learning environments was the final theme, and it was divided into sub-categories for the different characteristics that the students believed would improve their STEM learning environments. For the first sub-category of Hands On, students discussed wanting more physical experiences during which they could create and play to learn about concepts. One child explained that being involved in the learning was far more effective than “just like, watching videos or writing stuff down”. The second sub-category of Environment was related to ideas that the students had for their physical environment, including having more opportunities for flexible seating, including beyond the classroom where they could have meeting spaces or gaming rooms. Some schools develop Makerspaces, laboratories or technology laboratories to support their students, and this idea connects with those spaces. The third sub-category of Choice contained points that the students made about being able to have more agency with their learning. They discussed selecting with whom they work, having a choice of presentation style and being able to co-construct the curriculum for projects. The fourth sub-category of Technology related primarily to having access to more technologies. Specifically, the students listed Minecraft, Micro:bits, bee bots, and more opportunities for making and testing that were related to technologies. The final sub-category of Peer Collaboration involved students discussing *how* they prefer to work together. This included larger learning spaces and having access to people beyond their own class, or even beyond the school. This could connect to other classes or year levels, or to special guests and excursions.

Overall, students indicated that they believed that their STEM learning environment was positive and that generally students felt engaged. Students were able to describe many positive characteristics that their teachers implemented to support their STEM education and described several changes that they believed would improve their learning environment. Because student perceptions and perceived preferred perceptions provide valid and valuable insights into high-quality STEM learning environments, seeking these perceptions from multiple contexts could provide further insights for improving the engagement and aspirations of students within STEM education. As Fraser (2012) states, it would be a positive step to change learning environments, where possible, to suit the preferred perceptions of our students.

## Discussion

The drive to improve STEM education to meet future workforce needs for Australia’s economic success is critical (Education Services Australia, 2018; Hudson et al., 2015; Office of the Chief Scientist, 2013). High-quality STEM education that develops STEM skills will be essential for unknown roles which are necessary for Australia’s future (Caplan et al., 2016; Honey et al., 2014; Marginson et al., 2013; Timms et al., 2018). Learning environment research has demonstsrated that student perceptions of their Classroom Emotional Climate and their interactions with their teacher can impact their academic achievement and motivation (Reyes et al., 2012) and, therefore, it is important to consider student perceptions when designing learning environments (Fraser, 2012).

Using a mixed-methods approach, a students were able to express their perceptions about their STEM learning environments, including their perceived preferred perceptions. This insight might assist in further understanding the characteristics of STEM learning environments that are conducive to high-quality education and student engagement as needed by industry.

Key findings from this study include that students within this context did not associate well with behavioural freedom and, in fact, preferred environments that have structure and also promote agency linked to curriculum and communication choices. Similarly, in a recent study, Koul et al. (2021) found that students had relatively positive perceptions of teacher control during STEM learning, with females scoring more highly. Similar conclusions were reached in other studies, such as research with middle-school students in which girls had more positive perceptions of order, involvement and organisation (Waxman & Huang, 1998).

Peer Collaboration was also an important aspect of STEM learning environments, with students having the opportunity to learn and grow with each other through problems, without simply being given answers by their teachers. This essential concept links closely with several of the World Economic Forum (2020) Top 15 Skills for 2025, potentially including Complex Problem-Solving (3), Critical Thinking and Analysis (4), and Emotional Intelligence (11).

Another key finding included Problem Solving, with students being given opportunities to trial a range of solutions to problems. Closely related to Peer Collaboration, in the sense that students liked how their teachers didn’t simply give them the answer, this characteristic also develops a number of the Top 15 Skills for 2025 (World Economic Forum, 2020), potentially including Analytical Thinking and Innovation (1), Complex Problem-Solving (3), Critical Thinking and Analysis (4), Resilience, Stress Tolerance and Flexibility (9), and Reasoning, Problem-Solving and Ideation (10).

Communication was a theme linked to noise control within the classroom and was related to opportunities to discuss problems, as well as methods for communicating knowledge. Interestingly, communication skills are not explicitly stated within the World Economic Forum (2020) Top 15 Skills for 2025, though they might sit within categories such as Leadership and Social Influence (6) or Persuasion and Negotiation (15). Students also explained that they would like more agency regarding choosing how to present their information, which would then also promote the development of skills such as Creativity, Originality and Initiative (5).

Time was an interesting key theme that was brought up by the students through the focus groups. It relates to several of the other key findings because, without enough time, problem solving are more difficult to implement effectively. As discussed previously, it could be hard for children to understand why teachers need to limit time within the classroom to ensure that they are balancing curriculum requirements and high-quality teaching.

The perceived preferred environments findings highlighted concepts such as hands-on learning, physical environments, choices relating to agency, the frequent use of technology, and varied opportunities to collaborate beyond the classroom. These potential changes to the learning environment would also promote the development of a range of the World Economic Forum (2020) skills, including but not limited to Active Learning and Learning Strategies (2), Technology Use, Monitoring and Control (7), and Technology Design and Programming (8).

The development of these skills within STEM learning environments is crucial, and the use of integrated approaches to develop these competencies through authentic contexts is an effective method (Nadelson & Seifert, 2017; Rosicka, 2016). Positive experiences with these disciplines will also assist in improving student attitudes and aspirations (Murphy et al., 2019), which can lead to further engagement with these fields. Therefore, this study utilised the extensively researched field of learning environments to measure student perceptions of their context, to identify positive aspects of high-quality STEM learning environments, and identify potential deterrents to engagement.

## Conclusion

This research is significant in that it measured student perceptions of their STEM learning environment to determine how these perspectives impacted their engagement within their context. Additionally, we collected student perceived preferred characteristics of STEM learning environments and identified potential ways to improve engagement. This involved the functional validation of a questionnaire for upper-primary classrooms that can be utilised by other researchers and schools to assess students’ perceptions within other contexts.

The research also identified a potential deterrent which could negatively impact student perceptions of STEM education. Relating to the Student/Responsibility Freedom scale of the questionnaire and discussed multiple times within the semi-structured focus groups, it was shown that the students did not associate positively with freedom that related to behaviour issues and a lack of structure. As a child’s perception is their reality, it is crucial that we take into consideration their thoughts, feelings and attitudes when designing learning environments.
